# Expression of HIF-1α and Genes Involved in Glucose Metabolism Is Increased in Cervical Cancer and HPV-16-Positive Cell Lines

**DOI:** 10.3390/pathogens12010033

**Published:** 2022-12-25

**Authors:** Víctor D. Priego-Hernández, Adán Arizmendi-Izazaga, Diana G. Soto-Flores, Norma Santiago-Ramón, Milagros D. Feria-Valadez, Napoleón Navarro-Tito, Hilda Jiménez-Wences, Dinorah N. Martínez-Carrillo, Eric G. Salmerón-Bárcenas, Marco A. Leyva-Vázquez, Berenice Illades-Aguiar, Luz del C. Alarcón-Romero, Julio Ortiz-Ortiz

**Affiliations:** 1 Laboratorio de Biomedicina Molecular, Facultad de Ciencias Químico Biológicas, Universidad Autónoma de Guerrero, Av. Lázaro Cárdenas S/N, Ciudad Universitaria, Colonia La Haciendita, Chilpancingo C.P. 39090, Guerrero, Mexico; 2 Laboratorio de Biología Celular del Cáncer, Facultad de Ciencias Químico Biológicas, Universidad Autónoma de Guerrero, Av. Lázaro Cárdenas S/N, Ciudad Universitaria, Colonia La Haciendita, Chilpancingo C.P. 39090, Guerrero, Mexico; 3 Laboratorio de Investigación Clínica, Facultad de Ciencias, Químico Biológicas, Universidad Autónoma de Guerrero, Av. Lázaro Cárdenas S/N, Ciudad Universitaria, Colonia La Haciendita, Chilpancingo C.P. 39090, Guerrero, Mexico; 4 Laboratorio de Investigación en Biomoléculas, Facultad de Ciencias Químico Biológicas, Universidad Autónoma de Guerrero, Av. Lázaro Cárdenas S/N, Ciudad Universitaria, Colonia La Haciendita, Chilpancingo C.P. 39090, Guerrero, Mexico; 5 Departamento de Biomedicina Molecular, Centro de Investigación y de Estudios Avanzados del Instituto Politécnico Nacional, Ciudad de Mexico 07360, Mexico; 6 Laboratorio de Investigación en Citopatología e Histoquímica de la Facultad de Ciencias Químico Biológicas, Universidad Autónoma de Guerrero, Chilpancingo C.P. 39090, Guerrero, Mexico

**Keywords:** HPV 16, HIF-1α, glucose metabolism, metabolic reprogramming, cervical cancer

## Abstract

Cervical cancer (CC) is the most common cancer in women in the lower genital tract. The main risk factor for developing CC is persistent infection with HPV 16. The E6 and E7 oncoproteins of HPV 16 have been related to metabolic reprogramming in cancer through the regulation of the expression and stability of HIF-1α and consequently of the expression of its target genes, such as *HIF1A* (HIF-1α), *SLC2A1* (GLUT1), *LDHA*, *CA9* (CAIX), *SLC16A3* (MCT4), and *BSG* (Basigin or CD147), which are involved in glucose metabolism. This work aimed to evaluate the expression of HIF-1α, GLUT1, LDHA, CAIX, MCT4, and Basigin in patient samples and CC cell lines. To evaluate the expression level of *HIF1A, SLC2A1, LDHA, CA9, SLC16A3*, and *BSG* genes in tissue from patients with CC and normal tissue, the TCGA dataset was used. To evaluate the expression level of these genes by RT-qPCR in CC cell lines, HPV-negative (C-33A) and HPV-16-positive (SiHa and Ca Ski) cell lines were used. Increased expression of *HIF1A, SLC2A1, LDHA, SLC16A3*, and *BSG* was found in Ca Ski and *CA9* in SiHa compared to C-33A. Similar results were observed in CC tissues compared to normal tissue obtained by bioinformatics analysis. In conclusion, the expression of HIF-1α, GLUT1, LDHA, CAIX, MCT4, and BSG genes is increased in CC and HPV-16-positive cell lines.

## 1. Introduction

Cervical cancer (CC) is the fourth leading cause of death in women worldwide, with approximately 604,127 new cases and 341,831 deaths annually [1]. Human papillomavirus 16 (HPV 16) is present in more than 50% of cases of CC [2]. The oncogenicity of HPV results mainly from the action of E6 and E7 oncoproteins. E6 promotes carcinogenesis by inducing the degradation of the tumor suppressor protein p53 and activating the PI3K/AKT/mTOR [3]. In contrast, E7 contributes to carcinogenesis by inducing the retinoblastoma tumor suppressor protein (pRb) degradation by releasing the cell cycle transcription factor E2F1 [4]. Several studies have revealed the association between HIF-1α overexpression and worse prognosis in patients with this type of cancer [5]. In different HPV-16-positive cancers, other active HIF1-regulated genes are overexpressed, which encode proteins that play an essential role in immortalization [6,7], cell proliferation, metastasis, and metabolic reprogramming [8].

Metabolic reprogramming is an essential feature of cancer. Tumor cells reprogram their metabolism through a process known as the Warburg effect; this process is defined as the ability of cells to have high rates of glycolysis for the generation of ATP and precursors of different biomolecules independent of O_2_ [5,9]. The active HIF1 transcription factor is a master transcription factor of genes in response to hypoxia [10]. Active HIF1 is a heterodimer consisting of two subunits: HIF-α, which is induced by hypoxia and has three isoforms: HIF-1α, HIF-2α, and HIF-3α; and the HIF-1β subunit, which is constitutively expressed [10]. The transcription factors, such as the c-Myc-MAX heterodimer, the ISGF3 complex, composed of STAT1/STAT2/IRF9, STAT3, NF-κB, and even active HIF1 itself, bind to the promoter region of the HIF-1α gene to activate the initiation of its transcription [11]. Active HIF1 binds to the promoters of genes containing the 5′-RCGTG-3′ sequence, known as hypoxia response elements (HREs) [12]. Together with the coactivators CBP and p300 [13], they can regulate the expression of more than 70 genes, including some involved in glucose metabolism, such as HIF-1α, GLUT1, LDHA, CAIX, MCT4, and BSG (Basigin) [12,14]. HIF-1 upregulates the expression of *GLUT1* and *GLUT3* genes, both necessary for glucose uptake, and enhances lactate production by the enzyme lactate dehydrogenase A (LDHA), thereby decreasing intracellular pH. This mechanism regulates intracellular acidosis and is essential for maintaining homeostasis in the carbonic-anhydrase-IX (CAIX)-dependent mechanism [15]. Together with MCT4 and BSG, they facilitate the release of lactate and H+ to the extracellular milieu to neutralize intracellular acidosis in a HIF-1-dependent manner [16], which is associated with a hyperglycolytic and acid-fast phenotype in cancer [14,17,18] and the release of lactate into the extracellular milieu [19]. It has been shown in cancer in vitro that MCT4 is regulated by HIF1, unlike MCT1 and MCT2, because it has two HREs in its promoter [20].

It has been reported that under hypoxia conditions in CC, E7 is associated with increased expression of HIF-1α via ROS, ERK1/2, and NF-κB [2,11]. In turn, E6 positively regulates HIF-1α, preventing its ubiquitination by VHL and subsequent degradation via proteasome [21]. Thus, E7 and E6 could indirectly promote increased glycolysis and resistance to intracellular pH changes through increased expression of HIF-1α and its target genes, such as GLUT1, LDHA, CAIX, MCT4, and BSG [3,9,22]. Moreover, the high glucose levels in blood have been associated with bad prognosis in CC, suggesting a potential relationship between CC and the metabolism of glucose [23]. However, the role of HPV 16 oncoproteins E6 and E7 in metabolic reprogramming is not entirely clear. 

Therefore, in this work, we evaluated the expression of active HIF1 target genes: HIF-1α, GLUT1, LDHA, CAIX, MCT4, and BSG in HPV-16-positive CC cell lines and biopsies of CC patients using data from The Cancer Genome Atlas (TCGA). Likewise, an overall survival analysis was performed using the Kaplan–Meier Plotter database. Increased expression of *HIF1A, SLC2A1, LDHA, SLC16A3*, and *BSG* was observed in cell lines and biopsies of patients with CC and was associated with poor survival prognosis. This information will contribute to a better understanding of the mechanisms that favor metabolic reprogramming in HPV-16-positive CC. 

## 2. Materials and Methods

### 2.1. Gene Expression Analysis in CC Samples Using the TCGA and HPA Datasets

For gene expression analysis of mRNA level in CC patient samples, data were obtained from The Cancer Genome Atlas (TCGA) dataset and the GEPIA database [24]. The total was n = 306 biopsies from patients with CC and n = 13 with normal tissue. Graphs showed the expression levels of HIF-1α, GLUT1, LDHA, CAIX, MCT4, and BSG. Expression was log_2_ transformed (TPM+1), differences were calculated using a one-way ANOVA test, and a *p*-value < 0.05 was considered statistically significant. The expression of these six genes compared to protein level was analyzed in the Human Protein Atlas (HPA) database [25].

### 2.2. Correlation Analysis

Correlation analysis between *HIF1A* expression and *SLC2A1, LDHA, SLC16A3*, and *BSG* expression was performed on CC samples from the TCGA dataset using the GEPIA database [24]. The correlation was calculated using Spearman and R2 coefficients; a value of *p* < 0.05 was considered statistically significant.

### 2.3. Cell Culture

C-33A, SiHa, and Ca Ski cell lines were cultured in DMEM (Dulbecco’s Modified Eagle’s Medium) supplemented with 10% fetal bovine serum (Gibco, Life Technologies, Grand Island, NY, USA), and 100 U/mL penicillin and 100 µg/mL streptomycin (Gibco, Life Technologies, Grand Island, NY, USA) were added. The cells were maintained at a temperature of 37 °C with an atmosphere of 5% CO_2_.

### 2.4. RNA Extraction

Total RNA extraction was performed using TriZol^®^ Reagent (Ambion^®^ by Life Technologies, Carlsbad, CA, USA), following the manufacturer’s instructions. Total RNA integrity was verified by 1.5% agarose gel electrophoresis. The concentration and purity of the RNA obtained were determined by spectrophotometry using the Nanodrop 2000c (Thermo Fisher Scientific Waltham, MA, USA).

### 2.5. Determination of Gene Expression in C-33A, SiHa, and Ca Ski Cells by RT-qPCR

Determination of gene expression in C-33A, SiHa, and Ca Ski cells was performed by RT-qPCR using the TaqMan^®^ RNA-to-Ct™ 1-Step Kit (4392938). Each 10 µL reaction contained 1 µL of total RNA (50 ng); 5.0 µL of TaqMan^®^ RT-PCR Mix reaction mix (2✕), which contains AmpliTaq Gold^®^ DNA Polymerase, UP (Ultra Pure), dNTPs (dATP, dCTP, dGTP, dTTP, and dUTP), ROX™ passive reference and optimized buffer components; 0.25 µL of TaqMan^®^ RT Enzyme Mix (40×) containing: ArrayScript™ UP Reverse Transcriptase and RNase inhibitor; 0.5 µL of HIF1-α (ID: HS0015153153_M1), GLUT1 (ID: HS00892681_m1), LDHA (ID: HS01378790_g1), MCT4 (ID: HS00358829_m1), CAIX (ID: HS00154208_m1), and BSG (ID: HS00936295_m1) probes, respectively. The probe used as endogenous was GAPDH (ID: HS99999905_05), with 3.25 µL of nuclease-free H_2_O. Thermal cycling conditions were as follows: 48 °C for 15 min for retrotranscription, 95 °C for 10 min to activate DNA polymerase, 40 cycles of 95 °C for 15 s for cDNA denaturation, 60 °C for 1 min for alignment and extension on the 7500 Fast System real-time PCR system (Applied Biosystems, South San Francisco, CA 94080, USA). Gene expression was expressed as averages ± SD and determined using the 2^−ΔΔCT^ method [26].

### 2.6. Overall and Relapse-Free Survival Analyses

Overall Survival (OS) analyses were performed from the TCGA dataset using the Kaplan–Meier Plotter database (https://kmplot.com/analysis/ accessed on 20 June 2022) [27]. Survival curves were estimated using the Kaplan–Meier estimator. Survival curves were compared with the log-rank test. Data were analyzed for CC using the pan-cancer expression option. 304 patients were analyzed from the database repository.

### 2.7. Statistical Analysis

The analysis of data obtained from the cell lines was performed by multivariate analysis using ANOVA. Post hoc Tukey tests considered a value *p* < 0.05 as statistically significant. Survival analyses with a *p* < 0.05 were considered statistically significant using the log-rank test. A *p* < 0.01 was considered statistically significant for expression analyses in patient samples.

## 3. Results

### 3.1. HIF1A, SLC2A1, LDHA, CA9, SLC16A3, and BSG Expression Is Increased in Samples from Patients with CC

**Figure 1:**
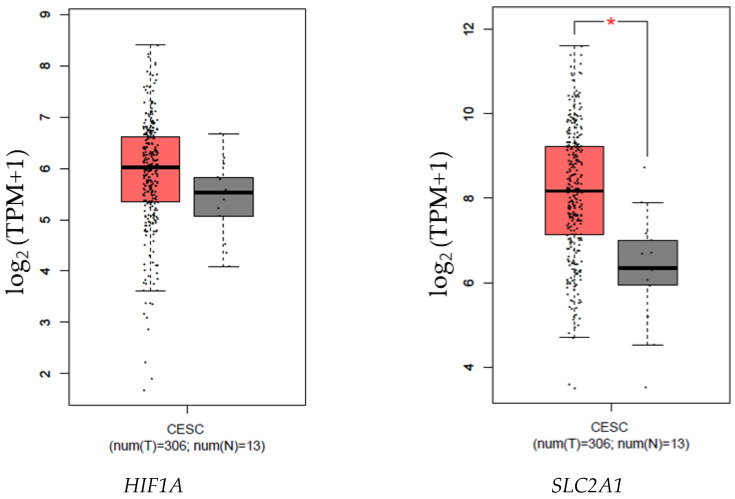

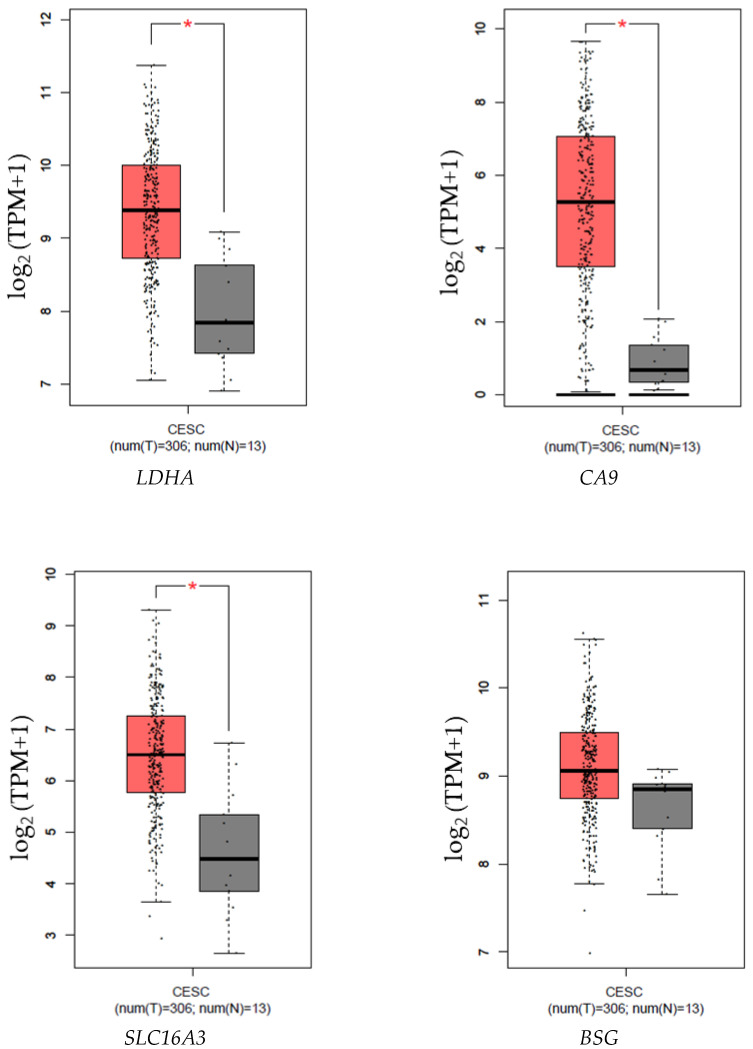
Expression of *HIF1A* and its target genes *SLC2A1, LDHA, CA9, SLC16A3*, and *BSG* in biopsies of patients with CC (T) (red box) and normal tissue (N) (grey box), obtained from the TCGA dataset, is shown. * *p* < 0.01. Expression was log_2_ transformed (TPM+1).

Metabolic reprogramming is a key feature in cancer progression. For this reason, the expression of *HIF1A, SLC2A1, LDHA, CA9, SLC16A3*, and *BSG* genes was analyzed in samples from patients with CC and normal tissue using the TCGA and GEPIA datasets [24]. The expression level of the genes of interest was obtained in 13 samples from normal tissue and 306 from patients with CC. Increased expression levels of *SLC2A1, LDHA, CA9,* and *SLC16A3* were observed in CC. The differences were statistically significant compared to normal tissue samples. We also found that HIF-1α and BSG expression levels increased in CC compared to normal tissue (Figure 1). To confirm these results, we compared the expression of six genes to the protein level in tissue samples from the HPA dataset, and we found similar results (Figure S1).

### 3.2. High Expression of HIF1A Correlates with Increased Expression of SLC2A1, LDHA, CA9, SLC16A3, and BSG in CC

*SLC2A1, LDHA, CA9, SLC16A3*, and *BSG* are transcriptional targets of the active transcription factor HIF1. To evaluate whether increased expression levels of HIF-1α correlate with increased expression levels of the target genes of active HIF1, a correlation analysis was performed between expression levels of HIF-1α and *SLC2A1, LDHA, CA9, SLC16A3*, and *BSG* in the GEPIA database (Table 1). It was observed that expression of *SLC2A1, LDHA, CA9*, and *SLC16A3* shows a positive correlation with *HIF1A* expression, i.e., when *HIF1A* expression levels increase, *SLC2A1, LDHA, CA9,* and *SLC16A3* expression levels also increase; in all cases the data were statistically significant. However, in the case of *BSG*, when *HIF1A* levels are low, *BSG* expression levels increase, although the data were not statistically significant. These data suggest that in CC, the high expression of *SLC2A1, LDHA, CA9, SLC16A3*, and *BSG* is related to the high expression of the HIF-1α subunit of the active HIF1 complex.

### 3.3. mRNA Expression of HIF1A, SLC2A1, LDHA, CA9, SLC16A3, and BSG Is Increased in CC Cell Lines

**Figure 2:**
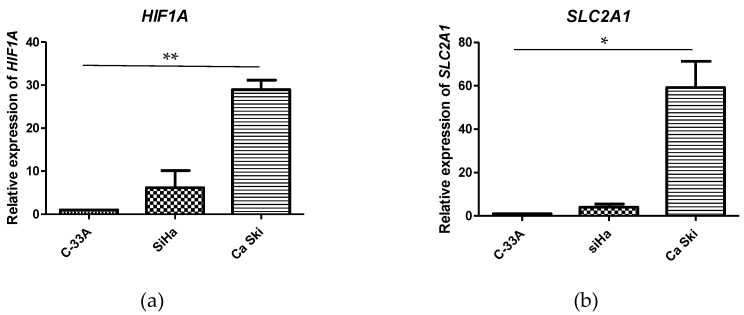

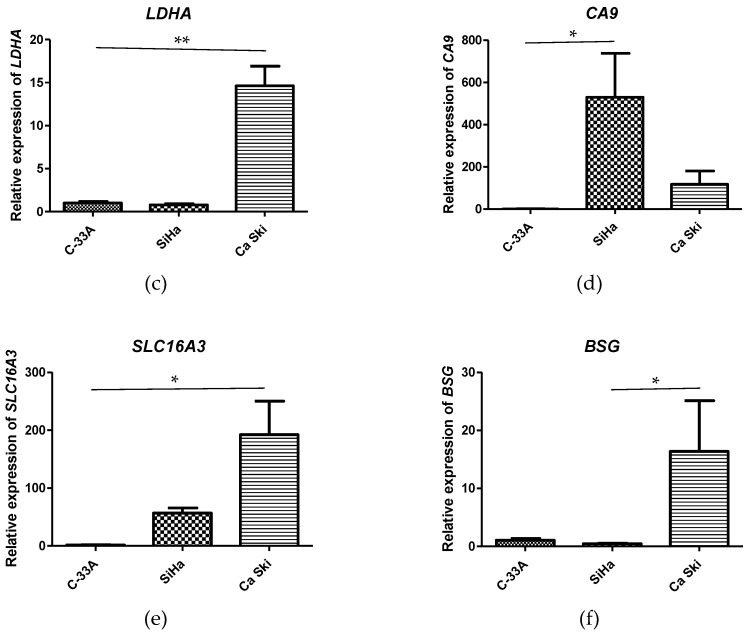
Relative expression of genes involved in metabolic reprogramming. The relative expression level of *HIFA1* (**a**), *SLC2A1* (**b**), *LDHA* (**c**), *CA9* (**d**), *SLC16A3* (**e**), and *BSG* (**f**) in the SiHa and Ca SKi cell compared to C33-A. A value of *p* < 0.05 was considered statistically significant through a one-way ANOVA test and using mean and standard error. Data were measured in three independent experiments in triplicate in RT-qPCR and calculated by the 2^−ΔΔCT^ method. Expression of the six transcripts was normalized to endogenous GAPDH. Relative expression levels were analyzed in GraphPad Prism software. * *p* < 0.05; ** *p* < 0.001.

To determine whether, as in patient samples, the expression levels of HIF-1α and its target genes *SLC2A1, LDHA, CA9, SLC16A3*, and *BSG* are increased in CC cell lines, their expression was evaluated in the SiHa and Ca Ski (HPV-16-positive) and C-33A (HPV-negative) cell lines. The results obtained show that the mRNA expression level of *HIF1A, SLC2A1, LDHA, CA9, SLC16A3*, and *BSG* increases in the Ca Ski cell line compared to C-33-A. However, a higher increase in CAIX was observed in SiHa compared to Ca Ski cells. Furthermore, in SiHa cells, an upregulation in the expression of HIF-1α and *SLC16A3* was observed compared to C-33A. On the other hand, a slight decrease in *LDHA* and *BSG* expression was observed in SiHa compared to C-33A; however, this decrease was not statistically significant (Figure 2). These results suggest that HPV 16 is involved in the overexpression of *HIF1A*, which in turn induces the overexpression of *SLC2A1, LDHA, CA9, SLC16A3*, and *BSG* involved in metabolic reprogramming in CC.

### 3.4. High Expression of HIF1A, SLC2A1, LDHA, CA9, and SLC16A3 Correlates with Lower Survival in CC

**Figure 3:**
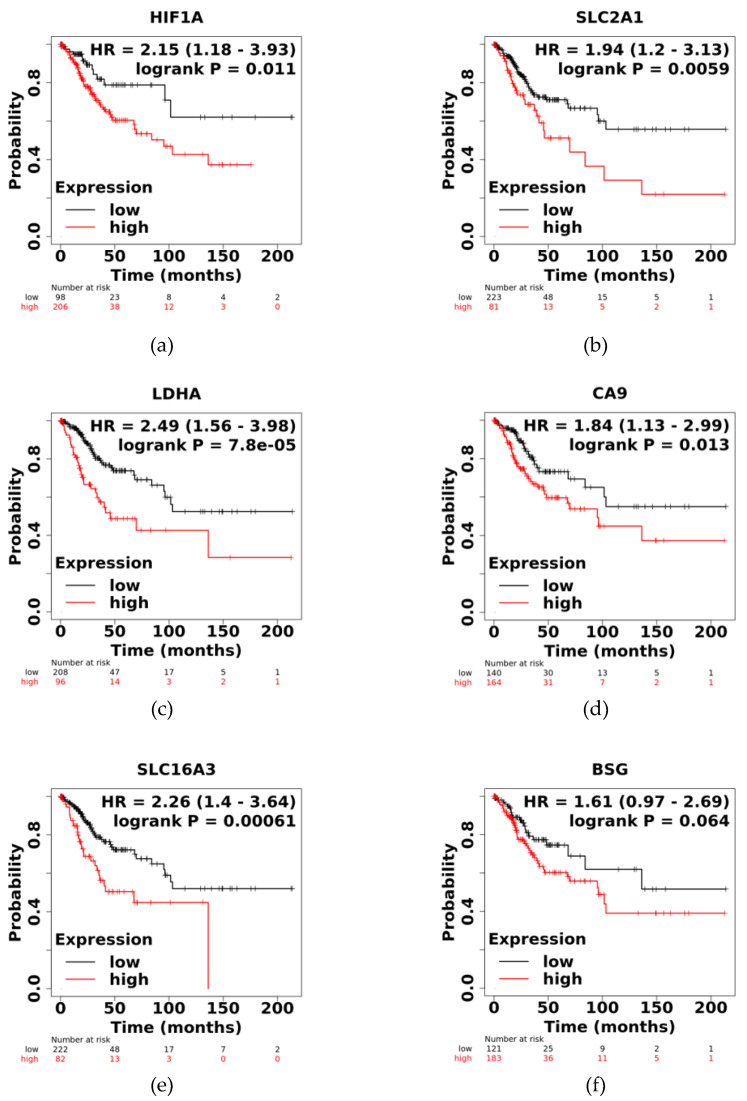
Overall survival analysis. Kaplan–Meier curve of overall survival by expression of *HIF1A* (**a**), *SLC2A1* (**b**), *LDHA* (**c**), *CA9* (**d**), *SLC16A3* (**e**), and *BSG* (**f**) in patients with CC. The data show the probability of survival for 200 months, the time during which the levels of the transcripts were studied. The lines in red show high levels, and the gray color shows low levels of gene expression. Numbers below the plots indicate the number of patients during baseline, 50, 100, 100, 150, and 200 months of expression analysis. *p <* 0.05 were considered statistically significant.

Here, we found that the expression of *HIF1A*, *SLC2A1*, *LDHA*, *CA9*, *SLC16A3*, and *BSG* genes is increased in CC samples (Figure 1) and in HPV-16-positive CC cell lines (Figure 2). Furthermore, high expression of HIF-1α was observed to correlate with increased expression of *SLC2A1*, *LDHA*, *CA9*, and *SLC16A3* genes in CC patient samples (Table 1). To determine whether high expression of *HIF1A*, *SLC2A1*, *LDHA*, *CA9*, *SLC16A3*, and *BSG* genes is involved in survival in patients with CC, OS analyses were performed using the Kaplan–Meier Plotter database (https://kmplot.com/analysis/ accessed on 20 June 2022). High expression of *HIF1A, SLC2A1, LDHA, CA9,* and *SLC16A3* genes was associated with shorter OS in patients diagnosed with CC, and these differences were statistically significant (Figure 3).

## 4. Discussion

Under hypoxia conditions in CC, it has been observed that HPV 16 oncoproteins E6 and E7 positively regulate HIF-1α. On the one hand, E7 promotes its gene expression, and on the other hand, E6 prevents its ubiquitination by VHL and its subsequent degradation via proteasome [21]. Several studies show that active HIF1 regulates gene expression in different hallmarks of cancer. The deregulated genes are grouped into tumor suppressor genes and oncogenes. In CC, deregulation of the expression of several genes is a mechanism that promotes tumor development and progression. These genes are known to code for proteins involved in processes such as metabolic reprogramming [9,22], angiogenesis [28], cell migration, invasion, and metastasis [29,30]. High expression of GLUT1, LDHA, and MCT4 proteins has been observed in biopsies from patients with invasive cervical cancer [31]. Additionally, LDHA inhibition has resulted in cell cycle inhibition and apoptosis in nasopharyngeal carcinoma [32]. It also suppresses cell migration, increases chemo- and radiosensitivity in cancer cells [33], induces cell cycle arrest in the G2/M phase, and activates the mitochondrial apoptosis pathway in CC cells [34]. On the other hand, there is evidence that *HIF1A* mRNA is overexpressed in CC [35] and laryngeal squamous cell carcinoma [36], while GLUT1 is overexpressed in CC [37] and colorectal cancer [38]. However, overexpression of *LDHA*, *CAIX*, *MCT4*, and *BSG* has only been reported in other types of cancer, but not in CC. *LDHA* overexpression has been reported in lung adenocarcinoma [39], *CAIX* in breast cancer [40] and oral squamous cell carcinoma [41], *MCT4* in bladder [42] and breast cancer [43], and *BSG* in acute myeloid leukemia [44]. In this work, the expression of *HIF1A*, *SLC2A1*, *LDHA*, *CA9*, *SLC16A3*, and *BSG* was evaluated in tissue from patients with CC and normal tissue using the TCGA dataset and in HPV-negative (C-33A) and HPV-16-positive (SiHa and Ca Ski) cell lines.

The expression of *HIF1A*, *SLC2A1*, *LDHA*, *CA9*, *SLC16A3*, and *BSG* was found to be increased in 306 samples from patients with CC compared with 13 samples of normal tissue. The increased expression of *SLC2A1*, *LDHA*, *CA9*, and S*LC16A3* is statistically significant. However, the increase in *HIF1A* and *BSG* expression was not statistically significant. Increased expression was found for *SLC2A1* [45], *LDHA* [46], *CA9*, *SLC16A3*, *BSG* [47] and HIF-1α [35,48]. Active HIF1 plays an important role in metabolic reprogramming in cancer by activating the transcription of genes encoding proteins involved in glucose metabolism, which promotes glucose uptake, conversion of pyruvate to lactate, pyruvate detour from mitochondria, and selective mitochondrial autophagy [49]. Active HIF1 regulates the expression of GLUT1, LDHA, CAIX [14], MCT4, and CAIX [12]. It can also bind to the promoter region of HIF-1α and promote its expression [11]. HIF-1α is the regulatory subunit in the formation of active HIF1, it is expressed under hypoxic conditions, and its expression is related to the Warburg effect in cancer [50]. These data suggest that the increased expression of *HIF1A*, *SLC2A1*, *LDHA*, *CA9*, *SLC16A3*, and *BSG* could be related to HIF1 activation triggered by increased *HIF1A* expression. These data were confirmed in the correlation analysis, where a positive correlation between the expression of HIF-1α and *SLC2A1*, *LDHA*, *CA9*, and *SLC16A3* was observed. Interestingly, when *HIFA1* expression is increased, *SLC2A1*, *LDHA*, *CA9*, and *SLC16A3* expression is also increased. A negative correlation was observed between *HIF1A* expression and *BSG* expression, although the data were not statistically significant.

On the other hand, in The Human Protein Atlas (https://www.proteinatlas.org/ accessed on 11 November 2022), gene expression data for *HIF1A*, *SLC2A1*, *LDHA*, *SLC16A3*, and *BSG* were found in 69 human cell lines. Interestingly, no data related to *CA9* expression were found in SiHa, HeLa, and HaCaT cell lines (Figure S2). The data found show an increase in the expression of *HIF1A*, *SLC2A1*, and *SLC16A3* transcripts, a slight increase in *BSG* expression, and a decrease in *LDHA* expression in the SiHa cell line, while in the HeLa cell line (HPV-18-positive), only increased expression of *SLC16A3* was observed, compared to the immortalized human keratinocyte cell line (HaCaT). These data suggest that high-risk HPV could regulate the expression of *HIF1A*, *SLC2A1*, *SLC16A3*, and *BSG* genes in these BESEbases, which are required to carry out metabolic reprogramming. Importantly, no reports on the expression levels of these transcripts in the C33-A and Ca Ski cell lines were found on this platform.

This work evaluated the expression of *HIF1A*, *SLC2A1*, *LDHA*, *CA9*, *SLC16A3*, and *BSG* transcripts in cervical cancer cell lines with and without HPV 16, compared to the HPV-negative C-33A tumor cell line, SiHa, with one to two integrated copies per cell of the HPV 16 genome, and Ca Ski with 600 integrated copies per cell of the HPV 16 genome. Increased expression of *HIF1A*, *SLC2A1*, *LDHA*, *CA9*, *SLC16A3*, and *BSG* transcripts was observed in the Ca Ski cell line compared to C-33A, with statistically significant differences in the expression of all genes evaluated, except *CA9*. In the SiHa cell line, there were only statistically significant differences in the expression of *CA9* compared to C-33A. There is also an increase in the expression of *HIF1A* and *SLC16A3* compared to C-33A; however, a slight decrease in *LDHA* and *BSG* was observed. When comparing the data obtained in this study with the data from the Human Protein Atlas, it is observed that there is a similar behavior in the expression of *LDHA*; however, it has been shown that in the SiHa cell line, E6 increases indirectly the LDHA expression via downregulation of miR-34a, which is a target of p53 [51]. On the other hand, the expression observed in the Ca Ski and C-33A cell lines was similar to that observed in the 306 CC samples and the 13 normal tissue samples reported in the TCGA dataset, in which the expression of the six genes evaluated was found to be increased with statistically significant differences in *SLC2A1*, *LDHA*, *CA9,* and *SLC16A3* (Figure 1), as in primary advanced uterine cervical carcinoma [35], human-papilloma-virus-type-16-positive and negative cervical cancer, [37] and cervical cancer [42].

These results support the theory that HPV 16 could be favoring the gene expression of *HIFA1* through E7 and the formation of active HIF1 through E6 and that, in turn, active HIF1 may be inducing the expression of its target genes, such as *HIF1A*, *SLC2A1*, *LDHA*, *CA9*, S*LC16A3*, and *BSG*. This effect could be affected by viral load, E6, and E7 variants, or at the stage of tumor progression. Importantly, SiHa and Ca Ski cells, in addition to being HPV-16-positive, show two different stages of cancer progression, as they were derived from primary cervical carcinoma and metastatic tumor cells, respectively [52]. Ca Ski is a cervical cancer cell line established from cells from metastasis in the mesentery of the small intestine and containing the integrated HPV 16 genome of about 600 copies per cell and the E-G131/G350 variants of E6 and E7-Prototype [53]. On the other hand, the SiHa cell line was established from primary uterine squamous cell carcinoma tissue. It contained the integrated HPV 16 genome of one to two copies per cell and the E-G350/C442 variants of E6 and E7-C645 [54,55], given that the E6 variants of HPV 16 have an oncogenic potential difference and induce the differential expression of several genes [56].These particularities of the cell lines could explain why, although the two cell lines contain HPV 16, in Ca Ski, there is a high expression of *HIF1A*, *SLC2A1*, *LDHA*, *SLC16A3*, and *BSG*, whereas in SiHa, there is a higher expression of *CA9*.

Changes in mRNA expression of various genes are often used to establish associations between gene transcription and disease stage. Previous studies have shown that high expression of *LDHA* is involved in cell proliferation and survival, migration, invasion, angiogenesis, and immune evasion in cancer, indicating that *LDHA* may be a potential prognostic marker and therapeutic target in cancer [7,57]. High *SLC2A1* expression and HPV 16 have been reported to be independent prognostic factors in patients with CC [37]. On the other hand, increased expression of MCT1 and MCT4 is generally associated with poor prognosis. MCT4 is overexpressed in different types of cancer, such as breast, bladder, colorectal, and CC cancers. Moreover, high expression of MCT4 is closely associated with increased expression of CAIX and BSG [16,22].

Regarding CAIX, its expression has been reported to regulate epithelial–mesenchymal transition and cell migration in CC [58]. In contrast, high expression of BSG has been correlated with radioresistance in the CC cell line SiHa [59]. Furthermore, it has been reported that shorter survival of patients in all types of breast cancer, especially in those with the triple-negative phenotype, is associated with high expression of HIF-1α [60], *CA9* [61], and *BSG* [62]; *LDHA* in lung adenocarcinoma [39]; *SLC16A3* in bladder cancer [42]; and *SLC2A1* in colorectal cancer [38]. Likewise, in this study, the overall survival analysis with Kaplan–Meier curves shows that high expression of *HIF1A*, *SLC2A1*, *LDHA*, *CA9*, *SLC16A3*, and *BSG* is associated with worse survival in patients with CC (Figure 3) in which HPV 16 is the leading etiological agent. All these data support the theory that a higher expression of the transcripts evaluated here is associated with HPV 16 and a worse prognosis in CC.

## 5. Conclusions

In conclusion, these results suggest that HPV 16 increases the expression of active HIF1 target genes, *HIF1A*, *SLC2A1*, *LDHA*, *CA9*, *SLC16A3*, and *BSG*, in the Ca Ski cell line and in patients with CC. On the other hand, the high expression of these genes is related to lower survival in patients with CC, denoting the importance of studying these genes and their possible use as prognostic biomarkers. This poor survival could be related to viral load, HPV 16 E6 and E7 variants, or stage of tumor progression; however, further studies are needed in this regard.

## Figures and Tables

**Table 1:** Correlation analysis between HIF-1α expression levels and *HIF1A*, *SLC2A1*, *LDHA*, *CA9*, *SLC16A3*, and *BSG* expression levels.

HIF-1α Target Genes	CESC (Cervical Squamous Cell Carcinoma and Endocervical Adenocarcinoma)	
	Tumor	
	*R*	*p*
*SLC2A1*	0.27	***
*LDHA*	0.41	***
*CA9*	0.14	**
*SLC16A3*	0.31	***
*BSG*	−0.26	0.66

Tumor, tissue correlation analysis TCGA. (**) *p* < 0.01, (***) *p* < 0.001. *R* (correlation) and *p* (*p*-value).

## Data Availability

No applicable.

## Supplementary Materials

The following supporting information can be downloaded at: https://www.mdpi.com/article/10.3390/pathogens12010033/s1, Figure S1: HIF-1α, GLUT1, LDHA, CA9, MCT4, and BSG protein expression increases in CC. Figure S2: HIF-1α, GLUT1, LDHA, MCT4, and BSG transcript expression in HaCaT, HeLa, and SiHa cervical cancer cell lines.
