# Supplementary material for: Expression of HIF-1α and Genes Involved in Glucose Metabolism Is Increased in Cervical Cancer and HPV-16-Positive Cell Lines
Source: Pathogens. 2022 Dec 25;12(1):33. doi: 10.3390/pathogens12010033 (PMC9865746; doi:10.3390/pathogens12010033)
Supplement: Supplementary file 1 [file pathogens-12-00033-s001.zip › pathogens-2063115-supplementary.pdf]

Supplementary S1

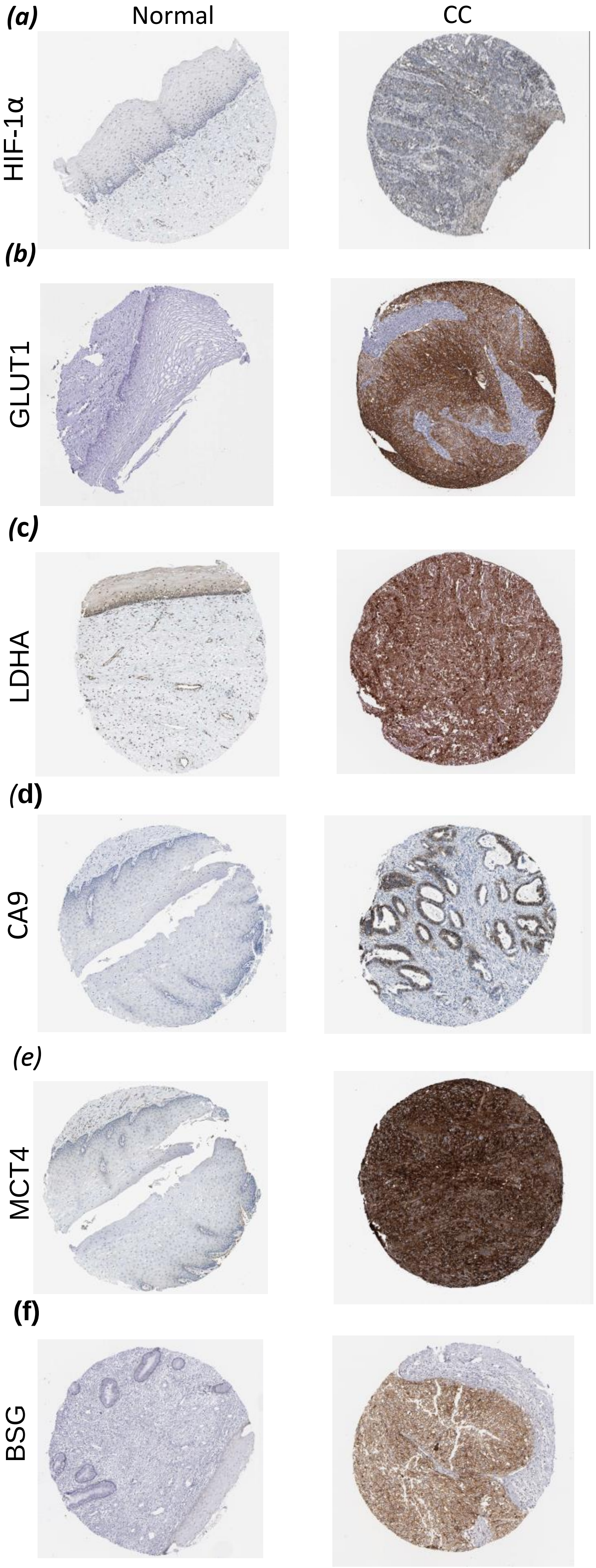

Figure S1. HIF-1α, GLUT1, LDHA, CA9, MCT4, and BSG protein expression increases in CC. The HIF-1α (a), GLUT1 (b), LDHA (c), CA9 (d), MCT4 (e), and BSG (f) expression was analyzed in normal (*left*) and CC (*right*) tissue samples from the Human Protein Atlas database. A representative image for each protein is showed.

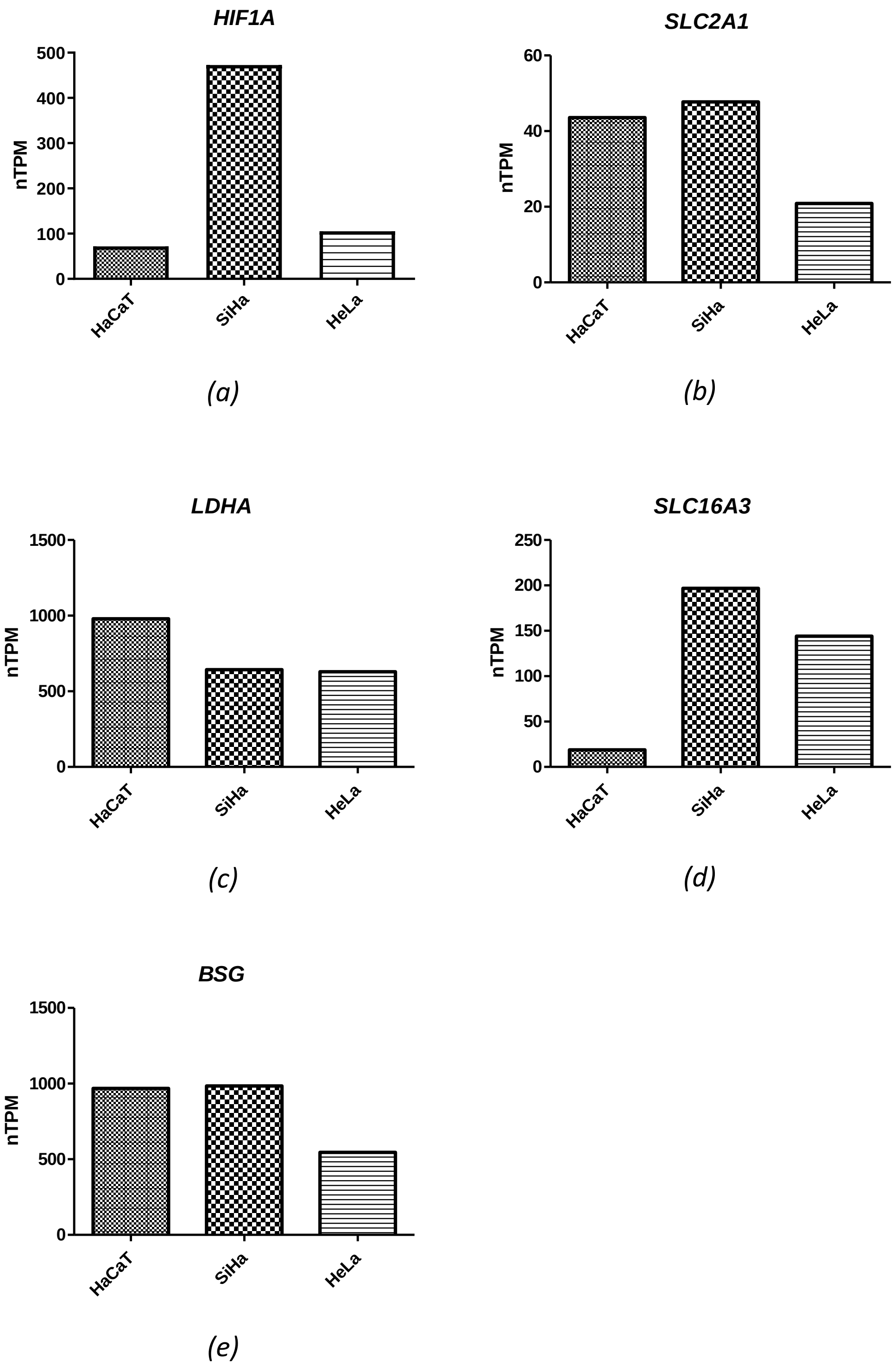

Figure S2. HIF-1α, GLUT1, LDHA, MCT4, and BSG transcript expression in HaCaT, HeLa, and SiHa cervical cancer cell lines. The data was reported in nTPM (Transcripts per million kilobases) obtained from the expression of the transcripts. Graphs were created using GraphPad Prism software. (a) HIF1A, (b) SLC2A1, (c) LDHA, (d) SLC16A3, and (e) BSG.
